# 3D Culture and Interferon-γ Priming Modulates Characteristics of Mesenchymal Stromal/Stem Cells by Modifying the Expression of Both Intracellular and Exosomal microRNAs

**DOI:** 10.3390/biology12081063

**Published:** 2023-07-28

**Authors:** Matteo Bulati, Alessia Gallo, Giovanni Zito, Rosalia Busà, Gioacchin Iannolo, Nicola Cuscino, Salvatore Castelbuono, Claudia Carcione, Claudio Centi, Gennaro Martucci, Alessandro Bertani, Maria Pia Baiamonte, Cinzia Maria Chinnici, Pier Giulio Conaldi, Vitale Miceli

**Affiliations:** 1 Research Department, IRCCS-ISMETT (Istituto Mediterraneo per i Trapianti e Terapie ad Alta Specializzazione), 90127 Palermo, Italy; mbulati@ismett.edu (M.B.); agallo@ismett.edu (A.G.); gzito@ismett.edu (G.Z.); rbusa@ismett.edu (R.B.); giannolo@ismett.edu (G.I.); ncuscino@ismett.edu (N.C.); scastelbuono@ismett.edu (S.C.); ccenti@ismett.edu (C.C.); baiamontemariapia@gmail.com (M.P.B.); pgconaldi@ismett.edu (P.G.C.); 2 Fondazione Ri.MED, 90128 Palermo, Italy; ccarcione@fondazionerimed.com (C.C.); cchinnici@fondazionerimed.com (C.M.C.); 3 Department of Anesthesia and Intensive Care, IRCCS-ISMETT (Istituto Mediterraneo per i Trapianti e Terapie ad Alta Specializzazione), 90127 Palermo, Italy; gmartucci@ismett.edu; 4 Thoracic Surgery and Lung Transplantation Unit, IRCCS-ISMETT (Istituto Mediterraneo per i Trapianti e Terapie ad Alta Specializzazione), 90127 Palermo, Italy; abertani@ismett.edu

**Keywords:** mesenchymal stromal/stem cells, microRNAs, MSC priming, IFN-γ priming, MSC spheroids, exosomes, MSC therapeutic properties, regenerative medicine

## Abstract

**Simple Summary:**

With the aim of improving the therapeutic potential of mesenchymal stromal/stem cells (MSCs), we analyzed miRNA expression to investigate the effects of priming on intracellular and exosome (EXO)-derived miRNAs of MSCs. We primed MSCs with 3D culture (3D MSCs) or IFN-γ treatment (γ-MSCs), and EXOs were isolated from the conditioned medium. The miRNA analysis revealed similar expression patterns in intracellular miRNAs among biological replicates, while we observed noticeable variability in EXO miRNAs released even with the same priming condition. Although the MSCs and their EXOs exhibited distinct miRNA profiles following each priming treatment, we found deregulated miRNAs in common between the two sample types. The gene ontology of the deregulated miRNAs obtained after priming showed that MSC and EXO-derived miRNAs were functionally associated with tissue repair/regeneration. Specifically, γ-MSCs, 3D MSC EXOs, and γ-MSC EXOs contained more enriched miRNAs related to immunomodulation compared with 3D MSCs. Moreover, compared with IFN-γ treatment, both cells and EXOs derived from the 3D culture had more enriched miRNAs targeting genes involved in angiogenesis. Our study demonstrates that both 3D culture and IFN-γ treatment are able to modify intracellular and exosomal miRNAs, and our findings might contribute to a better understanding of the molecular mechanisms underlying the miRNA-mediated beneficial effects of MSCs.

**Abstract:**

Mesenchymal stromal/stem cells (MSCs) have emerged as a therapeutic tool in regenerative medicine. Recent studies have shown that exosome (EXO)-derived microRNAs (miRNAs) play a crucial role in mediating MSC functions. Additionally, intracellular miRNAs have been found to regulate MSC therapeutic capacities. However, the molecular mechanisms underlying miRNA-mediated MSC effects are not fully understood. We used 3D culture and IFN-γ to prime/enhance the MSC therapeutic effects in terms of functional miRNAs. After priming, our analysis revealed stable variations in intracellular miRNA among the MSC biological replicates. Conversely, a significant variability of miRNA was observed among EXOs released from biological replicates of the priming treatment. For each priming, we observed distinct miRNA expression profiles between the MSCs and their EXOs. Moreover, in both types of priming, gene ontology (GO) analysis of deregulated miRNAs highlighted their involvement in tissue repair/regeneration pathways. In particular, the 3D culture enhanced angiogenic properties in both MSCs and EXOs, while IFN-γ treatment enriched miRNAs associated with immunomodulatory pathways. These findings suggest that 3D culture and IFN-γ treatment are promising strategies for enhancing the therapeutic potential of MSCs by modulating miRNA expression. Additionally, the identified miRNAs may contribute to understanding the molecular mechanisms underlying the miRNA-mediated therapeutic effects of MSCs.

## 1. Introduction

Cell therapies based on the use of mesenchymal stromal/stem cells (MSCs) have shown promising results in the field of regenerative medicine [1,2,3]. These therapies rely primarily on the paracrine action of soluble factors, including proteins and extracellular vesicles (EVs) such as exosomes (EXOs) [4,5]. EXOs enclose a plethora of functional molecules, such as growth factors, cytokines, chemokines, and microRNAs (miRNAs), with the latter considered key epigenetic modulators affecting the protein expression of target mRNAs [6,7,8]. Indeed, it has been shown that circulating miRNAs play a central role in the cellular communication regulating the phenotype of the target cells and represent a useful tool as biomarkers in various clinical conditions [9,10,11]. Interestingly, intracellular miRNAs have also been found to be crucial for the regulation of MSC phenotype and consequent behavior as a therapeutic tool [12,13]. Therefore, miRNAs are considered key mediators of some MSC effects, including immunomodulation, angiogenesis, and tissue regeneration [6,14,15,16]. The intricate regulation of these pathways suggests the potential involvement of both intracellular miRNAs, which have the capacity to modulate MSC phenotype, and EXO miRNAs, which may play a role in mediating the paracrine actions of MSCs. However, the molecular mechanisms underlying the miRNA-mediated therapeutic effects of MSCs are still not fully understood. There is a need for a deeper understanding of the role of both intracellular and exosomal miRNAs in order to understand the mechanisms underlying MSC-mediated therapeutic effects and their potential application in diverse regenerative processes.

Increasing evidence supports the hypothesis that the MSC therapeutic potential can be modulated by preconditioning (MSC priming) through various strategies, including cytokine treatments, as well as growing MSCs under specific three-dimensional (3D) culture conditions [5,17,18]. In these cases, the production of MSC factors can be redirected toward increased therapeutic efficacy [5,19]. Studies have shown that EXOs derived from primed MSCs are functionally more effective in the regulation of both immunomodulation and angiogenesis [5,20,21].

In this study, we aimed to identify the most promising priming strategies that lead to the enrichment of functional miRNAs in MSCs. To this end, we investigated whether specific priming methods may be able to modify both intracellular and EXO miRNAs, ultimately improving their therapeutic effects. We analyzed miRNA expression patterns in both human amnion-derived MSCs (hAMSCs) primed through 3D culture (3D hAMSCs) or IFN-γ treatment (γ-hAMSCs) and their EXOs isolated from the conditioned medium. Our comparative study identified the 3D culture as well as the treatment of hAMSCs with IFN-γ as promising priming strategies to improve the MSC therapeutic potential in terms of functional miRNAs. In particular, following priming, hAMSCs and their EXOs showed different miRNA profiles related to the specific treatment/condition under which the cells and their EXOs were produced. These results might help to understand the molecular mechanism underlying the miRNA-mediated therapeutic effects of MSCs and might provide functional miRNA biomarkers of both hAMSCs and EXOs for the development of appropriate therapies to treat specific pathological conditions.

## 2. Materials and Methods

### 2.1. Isolation of Mesenchymal Stromal/Stem Cells from Human Amniotic Membrane

We isolated MSCs from human placenta of healthy donors within 6 h of birth. All procedures were approved by IRCCS ISMETT’s Institutional Research Review Board (IRRB, project identification code: IRRB/39/20). Before obtaining the placenta, the donor signed a written informed consent. To isolate hAMSCs, the amnion membrane was cut into small parts, and each fragment was decontaminated under sterile conditions in three different solutions: phosphate buffered saline (PBS) solution 1 (supplemented with 2.5% Esojod, Esoform, Rovigo, Italy) for 2–3 s; PBS solution 2 (supplemented with 500 U/mL penicillin, 500 mg/mL streptomycin and 12.5 mg/mL amphotericin B, Thermo Fisher Scientific, Waltham, MA, USA, and 1.87 mg/mL cefamezin, Pfizer, Milan, Italy) for 3 min; and PBS solution 3 (supplemented with 100 U/mL penicillin and 100 mg/mL streptomycin, Thermo Fisher Scientific) for 5 min. The decontaminated fragments were first digested for 9 min at 37 °C in Hanks’ balanced salt solution (HBSS, Basel, Switzerland) containing 2.5 U/mL dispase (Corning, Corning, NY, USA) and then digested in a solution containing 0.94 mg/mL collagenase A (Roche, Mannheim, Germany) and 20 mg/mL DNase (Roche, Mannheim, Germany) for 2.5 h at 37 °C. The obtained cells were filtered with both 100 µm and 70 µm cell strainers (BD Falcon, Franklin Lakes, NJ, USA) and then pelleted and resuspended in Roswell Park Memorial Institute (RPMI) 1640 medium supplemented with 10% fetal bovine serum (FBS) for cell counting. The isolated cells were cultured in polystyrene culture dishes (Corning) at 37 °C, 5% CO_2_ in Chang Medium (Irvine Scientific, Santa Ana, CA, USA). hAMSCs were phenotypically characterized with cytofluorimetric analysis for positive markers (CD90, CD73, and CD13) and negative markers (CD45 and HLA-DR) (BD Falcon) using a 16-color FACS Celesta SORP flow cytometer (BD Falcon).

### 2.2. Priming of hAMSCs with IFN-γ Treatment or by Growing as Spheroids and Conditioned Medium Preparation

To prime hAMSCs with IFN-γ, the cells were cultured with serum-free Dulbecco’s modified eagle medium (DMEM) in a monolayer (2D) for 48 h in the presence of 200 IU/mL of IFN-γ (Human IFN-γ1b premium grade, Miltenyi Biotec, Bergisch Gladbach, Germany). To obtain spheroids, hAMSCs were cultured in DMEM serum-free medium at 5% CO_2_ and 37 °C. In this case, hAMSCs were seeded in 6-well ultralow attachment plates (Corning) in a suspended state to allow for the formation of three-dimensional spheroids (3D). To collect the conditioned medium (CM), the media derived from the 2D cultures (with or without IFN-γ) or 3D cultures were collected after 48 h, centrifuged to remove the cell debris, and frozen at −80 °C until use.

### 2.3. Isolation and Characterization of Exosomes (EXOs)

The serum-free CM was used to collect EXOs. In particular, the CM obtained from each condition was centrifuged at 300× *g* for 10 min to remove the debris. Then, to further remove both cells and cell debris, the medium was centrifuged for 20 min at 16,500× *g* and finally ultracentrifuged at 120,000× *g* for 90 min at 4 °C to pellet the EXOs. The total protein content of the EXO samples was determined using the Micro BCA Protein Assay Reagent Kit (Thermo Scientific, Waltham, MA, USA) following the manufacturer’s instructions and using BSA as a standard. To characterize the EXOs, both the size distribution and concentration were determined with nanoparticle tracking analysis (NTA) in a NanoSight NS3000 (Malvern Instruments Ltd., Malvern, UK). The samples were diluted 1:500 with PBS to reach an optimal concentration for instrument linearity. Readings were taken on quintuplicate of 60 s at 25 frames per second at a camera level set to 16 and with manual monitoring of temperature. The data were then analyzed with NTA software version 3.1. (Build 3.1.54, Analitik LTD, Cambridge, UK).

### 2.4. Real-Time PCR Analysis of miRNAs with TaqMan Low Density Arrays

The expression of both intracellular and exosomal miRNAs was analyzed using a TaqMan array specific for human microRNA amplification according to the manufacturer’s instructions (Thermo Fisher Scientific). We isolated total RNA from both the hAMSCs and their EXOs, and the purity and quantification were determined using a NanoDrop spectrophotometer (Thermo Fisher Scientific). Afterwards, we used 300 ng of RNA to produce single-stranded cDNA using the high-capacity RNA-to-cDNA kit protocol (Thermo Fisher Scientific). Finally, the amplification of 754 human miRNAs was performed with a real-time PCR system (Thermo Fisher Scientific), and the obtained data were analyzed in SDS software v2.4. We utilized the equation 2^−DDCT^ and used the U6 as housekeeping to determine the expression level for each miRNA. Clustering was performed using ΔΔCt values. The relative expression values were z score transformed and used for the evaluation of differences among the primed and unprimed cells (2D cells without treatment, control). Hierarchical clustering of the miRNA fold change was performed using Euclidean distance algorithms in the Cluster 3.0 program, and a heat map was generated using the Java TreeView software 3.0.

### 2.5. Target Gene Prediction and Gene Ontology (GO) Analysis

The online database miRNA Enrichment Analysis and Annotation Tool 2.0 (miEAA 2.0) (https://ccb-compute2.cs.uni-saarland.de/mieaa/, accessed on 10 January 2023) [22] was used to examine functional enrichments in the biological process GO terms of the differentially expressed miRNAs. MiEAA integrates data from different sources, including Gene Ontology, miRBase, and miRTarBase, as well as other datasets, and is based on GeneTrail [23].

### 2.6. Statistical Analysis

All miRNA data are expressed as mean ± SD. The data from different treatments were compared using computerized statistical software with the ANOVA test. The data were further analyzed with a two-tailed Student’s *t*-test. The obtained *p*-values were adjusted for false discovery rate due to multiple testing correction [24]. Differences were considered statistically significant at *p* < 0.05. GraphPad Prism software package (version 6.0, La Jolla, CA, USA) was used for all analyses.

## 3. Results

### 3.1. Culture, Characterization, Spheroid Formation of hAMSCs, and EXO Isolation

**Figure 1:**
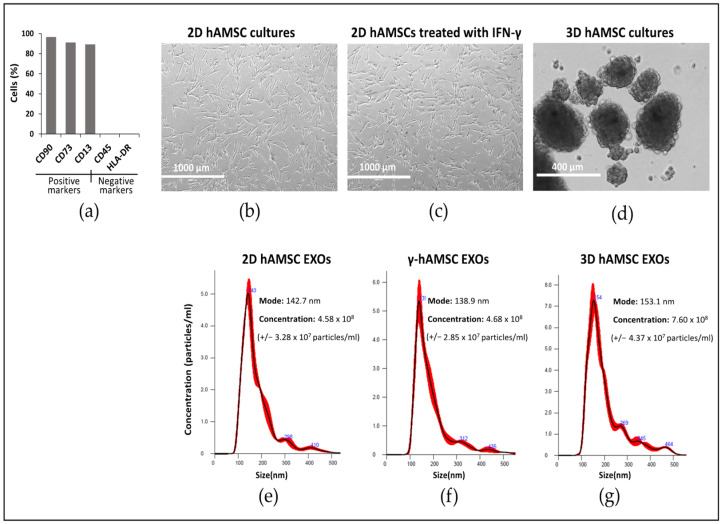
Human amnion-derived mesenchymal stem cells (hAMSCs) cultured as both a monolayer (2D culture) and spheroids (3D cultures). (**a**) Cytofluorimetric results of the positive and negative surface markers of hAMSCs. (**b**) Representative differential interference contrast (DIC) images of hAMSCs grown in a monolayer (2D cultures). (**c**) Representative DIC images of hAMSCs grown in a monolayer and treated with IFN-γ. (**d**) Representative DIC images of hAMSCs grown as spheroids (3D cultures). (**e**) Size and concentration (dilution 1:500) of exosomes isolated from the hAMSC 2D cultures. (**f**) Size and concentration (dilution 1:500) of exosomes isolated from the hAMSC 2D cultures treated with IFN-γ. (**g**) Size and concentration (dilution 1:500) of exosomes isolated from the hAMSC 3D cultures. The red lines in (**e**–**g**) describes the relationship between particle number distribution (left y-axis) and particle size (x-axis).

The hAMSCs were phenotypically characterized with flow cytometry for both positive markers (CD90, 98.4%; CD73, 90.8%; CD13, 89.80%) and negative markers (CD45, 0.1%; HLA-DR, 0.1%) (Figure 1a). The cells at passage two were grown in parallel in 2D culture without (2D hAMSCs) (Figure 1b) or with IFN-γ (γ-hAMSCs) (Figure 1c) and in a three-dimensional (3D) culture (3D hAMSCs) to allow for spontaneous aggregation into multicellular spheroids (Figure 1d). After priming, no differences in mortality were found between the treatments. After cultivating both 2D hAMSCs and primed cells (3D hAMSCs and γ-hAMSCs), we efficiently isolated the EXOs from each CM. In particular, we obtained EXOs with a diameter of 144 nm (average) and having a similar concentration for 2D hAMSC EXOs (2.29 × 10^11^ ± 3.28 × 10^7^ particles/mL) and γ-hAMSC EXOs (2.34 × 10^11^ ± 2.85 × 10^7^ particles/mL), while a higher concentration was obtained for 3D hAMSC EXOs (3.8 × 10^11^ ± 4.37 × 10^7^ particles/mL) (Figure 1e–g). Interestingly, the EXO concentration data were also confirmed with the analysis of the EXO protein amount, which was similar in 2D hAMSC EXOs and γ-hAMSC EXOs (8.409 µg/mL and 8.636 µg/mL, respectively) and higher in 3D hAMSC EXOs (13.953 µg/mL).

### 3.2. Differentially Expressed miRNAs (DEMs) in Primed hAMSCs

**Figure 2:**
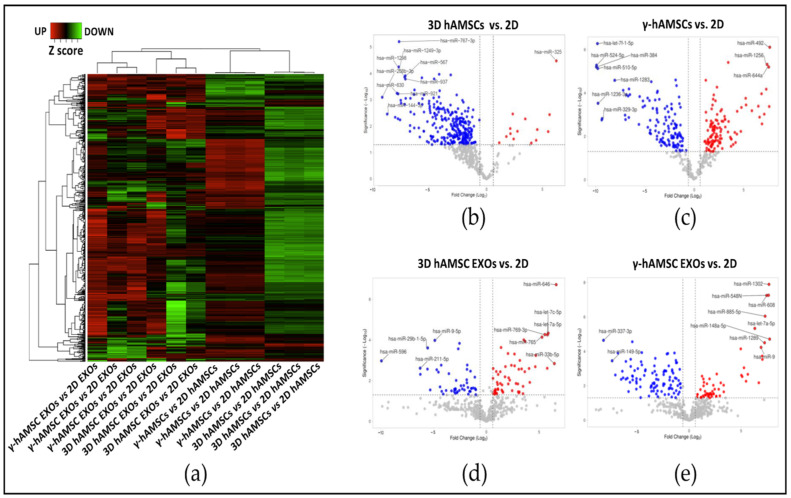
Cluster analysis to compare the fold changes (FC) in miRNA expression in primed hAMSCs and their EXOs. (**a**) Hierarchical clustering of z score transformed FC of miRNA in each treatment group compared to 2D untreated samples (control). (**b**) Volcano plot analysis (fold change > 1.5 and *p* < 0.05) of miRNA expression in the 3D culture of hAMSCs vs. control (2D culture). (**c**) Volcano plot analysis (fold change > 1.5 and *p* < 0.05) of miRNA expression in hAMSCs treated with IFN-γ vs. control (2D). (**d**) Volcano plot analysis (fold change > 1.5 and *p* < 0.05) of miRNA expression in EXOs derived from the 3D hAMSCs vs. control (2D). (**e**) Volcano plot analysis (fold change > 1.5 and *p* < 0.05) of miRNA expression in EXOs derived from hAMSCs treated with IFN-γ vs. control (2D). For all the volcano plots in the figure, red dots represent the up-regulated miRNAs in the experimental samples, while the blu dots represent the miRNAs up-regulated in the experimental controls.

The expression of 768 miRNAs was determined with real-time PCR analysis in both the hAMSCs and their EXOs. Multiple biological replicates were used, and the results revealed that, compared with the conventional 2D culture of hAMSCs, both priming treatments with IFN-γ and the 3D culture induced a stable gene expression variation among the replicates. On the other hand, an evident variability of the EXO miRNA expression was induced by both types of priming among the biological replicates (Figure 2a). Our statistical analysis, which employed a fold change threshold of greater than 1.5 and a *p*-value cutoff of less than 0.05, identified significant changes in miRNA expression in the different conditions. Specifically, in the 3D hAMSCs, 14 intracellular miRNAs were significantly up-regulated, while 368 intracellular miRNAs were significantly down-regulated (Figure 2b). In the γ-hAMSCs, 125 intracellular miRNAs were significantly up-regulated, and 130 intracellular miRNAs were significantly down-regulated (Figure 2c). Regarding the expression of EXO miRNAs, 57 miRNAs were found to be significantly up-regulated in the 3D hAMSC EXOs, while 107 miRNAs were significantly down-regulated (Figure 2d). In the γ-hAMSC EXOs, 146 miRNAs were significantly up-regulated, and 99 miRNAs were significantly down-regulated (Figure 2e).

**Figure 3:**
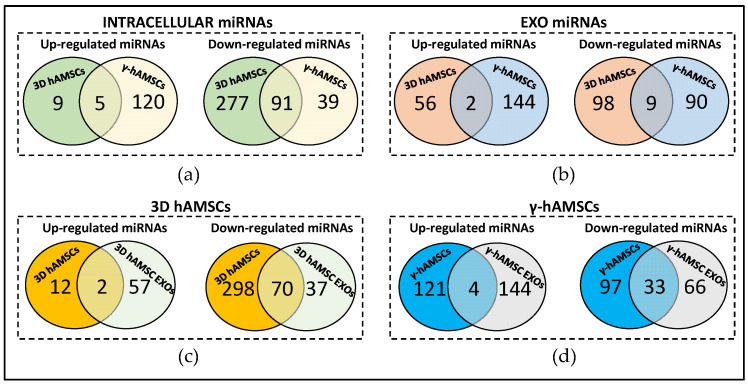
Venn diagram showing differentially expressed miRNAs between (**a**) 3D hAMSCs and hAMSCs treated with IFN-γ (γ-hAMSCs); (**b**) 3D hAMSC EXOs and γ-hAMSC EXOs; (**c**) 3D hAMSCs and 3D hAMSC EXOs; (**d**) γ-hAMSCs and γ-hAMSC EXOs.

Interestingly, among the intracellular DEMs, five miRNAs, miR-146b-5p, miR-212-3p, miR-1247-5p, miR-132-3p, and miR-194-5p, were up-regulated in both the 3D hAMSCs and γ-hAMSCs, while 91 down-regulated intracellular miRNAs were found in common in the same treatments (Figure 3a). In the EXOs, two miRNAs (miR-342-5p and miR-586) were up-regulated, and nine miRNAs were down-regulated commonly between the 3D hAMSC and γ-hAMSC EXOs (Figure 3b).

Moreover, for each priming treatment, the hAMSCs and EXOs had different miRNA expression profiles with the exception of a small number of up-regulated miRNAs in both the 3D hAMSCs (miR-212-3p and miR-132-3p) and IFN-γ-treated hAMSCs (miR-492, miR-133b, miR-188-3p, and miR-139-5p) (Figure 3c,d). Differently, a high number of miRNAs were significantly down-regulated in both the cells and EXOs in hAMSCs cultured in the 3D condition and IFN-γ treated hAMSCs (70 and 33, respectively) (Figure 3c,d).

### 3.3. Pathway Analysis of miRNA Targets (GO Biological Process)

**Figure 4:**
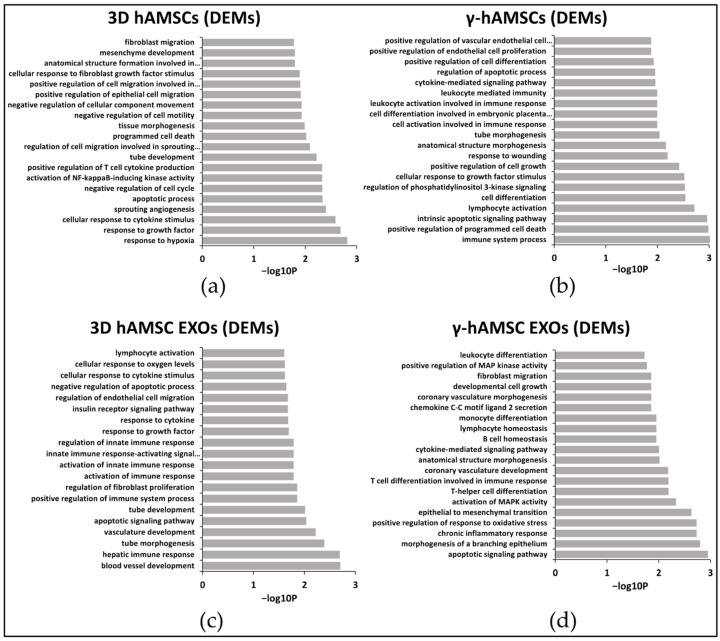
GO analysis of deregulated miRNAs (DEMs) in (**a**) hAMSCs grown as 3D spheroids (3D hAMSCs); (**b**) hAMSCs treated with IFN-γ (γ-hAMSCs); (**c**) exosomes (EXOs) derived from the 3D culture of hAMSCs; (**d**) EXOs derived from IFN-γ treated hAMSCs. Partial list of biological process enrichment analysis.

GO analysis was utilized to examine the target pathways of the top 100 significantly deregulated miRNAs. The results indicated distinct enrichment patterns for intracellular miRNAs in the 3D hAMSCs and IFN-γ treated hAMSCs as well as for miRNAs present in the hAMSC-derived EXOs. In particular, in the 3D hAMSCs, the DEMs were found to be significantly enriched in pathways related to cellular responses to hypoxia, growth factors, cytokines, angiogenesis, and apoptosis (top five ranked GO terms based on their *p*-values, Figure 4a). Conversely, in the IFN-γ treated hAMSCs, the DEMs were predominantly associated with immunomodulation and apoptosis processes (top five terms, Figure 4b). In the 3D hAMSC EXOs, the DEMs exhibited enrichment in terms related to angiogenesis processes, apoptosis, and hepatic immune responses (top five terms, Figure 4c). On the other hand, in the γ-hAMSC EXOs, the DEMs were enriched in several terms, including the “apoptotic signaling pathway”, “morphogenesis of a branching epithelium”, “chronic inflammatory response”, “positive regulation of response to oxidative stress”, and “epithelial to mesenchymal transition” (top five terms, Figure 4d).

We also detailed our GO analysis by analyzing the common DEMs across treatments/samples. In particular, among the five intracellular miRNAs up-regulated in both the 3D hAMSCs and γ-hAMSCs, GO analysis revealed that miR-212-3p, miR-132-3p, and miR-194-5p were enriched in numerous GO terms related to tissue regeneration/wound healing, such as “response to hypoxia”, “response to growth factor”, “cellular response to cytokine stimulus”, “apoptosis”, “cell cycle”, “tissue regeneration”, and “angiogenesis” (Table 1). In the hAMSCs primed with the 3D culture, hsa-miR-212-3p and hsa-miR-132-3p were also up-regulated in the EXOs. In the IFN-γ treated hAMSCs, regarding the four up-regulated miRNAs (miR-492, miR-133b, miR-188-3p, miR-139-5p) in common between the cells and EXOs, GO analysis revealed that those were enriched for “regulation of apoptosis”, “cell cycle regulation”, “tissue regeneration”, “angiogenesis”, and “regulation of immune system” terms (Table 1).

## 4. Discussion

MSCs possess strong immunoregulatory and angiogenic properties [25,26] and, therefore, have been widely tested in the field of regenerative medicine to treat numerous diseases [2,3,5,18,27,28,29]. In recent years, scientific evidence has revealed that MSC-derived products such as EXOs may contribute, at least in part, to MSC therapeutic effects [6,30,31,32,33]. Moreover, non-coding RNAs such as miRNAs have been considered the most functional factors that mediate some therapeutic functions of both MSCs and their EXOs [34,35,36,37]. Interestingly, it has been shown that different priming strategies are capable of improving the therapeutic properties of both MSCs and their products [5,17,38,39,40].

In our study, we investigated whether distinct priming strategies were able to modify both intracellular and exosomal miRNA expression, thus impacting MSC functions. To this end, we used MSCs derived from human amniotic placenta (hAMSCs) to analyze the expression of 768 miRNAs in both cells and their respective EXOs after priming with either 3D culture or IFN-γ treatment.

In the hAMSCs subjected to both priming treatments, we observed a stable variation in intracellular miRNA expression across biological replicates. However, notable variability in miRNA expression was observed in the EXO samples among the biological replicates (Figure 2a). This finding emphasizes the challenge of standardizing EXO production, which is a critical aspect for the development of cell-free, MSC-based therapies and highlights the need for standardization in the production of EXOs, as they are increasingly recognized as important mediators in MSC therapy.

As for the different priming approaches used, we observed that five intracellular miRNAs were up-regulated commonly between the two types of priming (Figure 3a). In particular, we found that miR-146b-5p, miR-212-3p, miR-1247-5p, miR-132-3p, and miR-194-5p were intracellularly overexpressed in both the 3D hAMSCs and γ-hAMSCs. Importantly, these miRNAs have been implicated in regulating the phenotype and therapeutic properties of MSCs. For example, miR-132-3p has been found to modulate the functional phenotype of MSCs by directly targeting the expression of ADAMTS-5. This interaction leads to an increase in the expression of SOX9, COL2A1, and ACAN, thereby promoting the chondrogenic differentiation of MSCs [41]. Furthermore, miR-146b-5p, miR-194-5p, and miR-212-3p have been associated with the therapeutic effects of MSCs [40,42,43,44]. Katakowski et al. showed that EXOs derived from miR-146b-transfected MSCs significantly reduced glioma growth in a rat model of brain tumor [42]. Sun et al. found that MSCs overexpressing miR-194-5p produced EXOs capable of delaying intervertebral disc degeneration in vitro [43]. Moreover, it has been shown that EXOs derived from MSCs overexpressing miR-212 were found to target ELF3 and suppress chondrocyte degeneration and inflammation in primary cells derived from patients with osteoarthritis [40].

Regarding miRNA expression in hAMSC-derived EXOs, we found two overexpressed miRNAs (miR-342-5p and miR-586) in common among the 3D and IFN-γ treated hAMSCs (Figure 3b). In these two miRNAs, miR-342-5p was found to be responsible for the therapeutic effects of MSCs, as demonstrated in different in vitro and in vivo models of distinct diseases. Xing et al. found that EXOs derived from MSCs exhibited protective effects on endothelial cells in an in vitro model of atherosclerosis, and this protective effect was mediated by the action of miR-342-5p, which targeted PPP1R12B [45]. Moreover, Liu and colleagues nicely proved that exosomal miR-342-5p derived from MSCs had the ability to mitigate acute kidney injury by inhibiting TLR9 in a sepsis mouse model [46].

We further analyzed miRNAs overexpressed in cells that are also packed into EXOs (Figure 3c,d). Our results showed that, in each priming treatment, miRNA expression was dissimilar in hAMSCs and their respective EXOs, as few miRNAs were overexpressed in common between the two types of samples (Figure 3c,d). In particular, in the 3D hAMSCs, we found that only two miRNAs (miR-212-3p and miR-132-3p) were overexpressed both in the cells and their EXOs (Figure 3c). As mentioned above, miR-212-3p has been shown to mediate some therapeutic effects of MSCs [40,44]. Moreover, it has been revealed that miR-132-3p delivered by MSC-derived EXOs was able to promote angiogenesis in myocardial infarction [47], alleviate synaptic dysfunction and cognitive decline in vascular dementia [48], and promote wound healing and skin reconstruction in an animal model of diabetic mouse [49]. In our investigation of γ-hAMSCs, we observed the overexpression of four specific miRNAs (miR-492, miR-133b, miR-188-3p, miR-139-5p) within the EXOs derived from these cells (Figure 3d). Previous studies provided evidence that EXOs derived from MSCs overexpressing miR-133b possess the capability to mitigate side effects in a rat model of intracerebral hemorrhage. These effects are attributed to the anti-apoptotic properties exhibited by miR-133b [50]. Li et al. demonstrated that EXOs overexpressing miR-188-3p effectively suppressed pyroptosis and offered protection in mice with induced Parkinson’s disease [51]. Furthermore, MSC-derived EXOs carrying miR-139-5p were shown to exert a tumor-suppressive role in bladder cancer [52].

**Figure 5:**
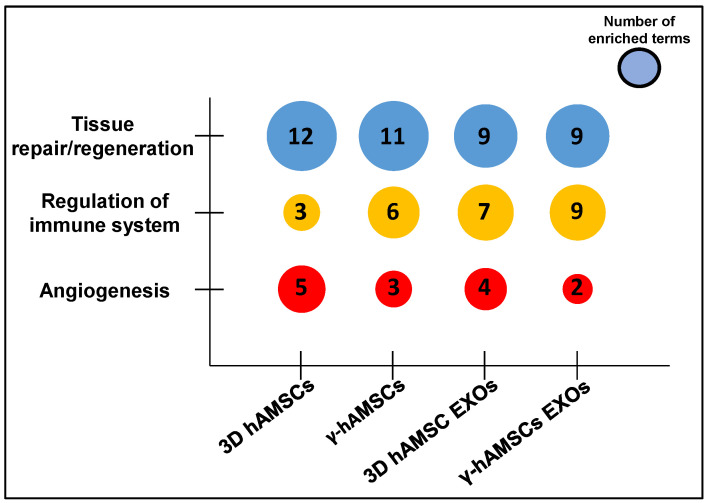
Bubble plot visualizing categories for GO-enriched terms of the DEMs in hAMSCs and their EXOs primed with either 3D culture or IFN-γ. Partial list of the 20 more significant enriched terms.

Overall, GO analyses revealed that the priming of hAMSCs with either 3D culture or IFN-γ treatment induces, in both cells and EXOs, an up-regulation of functional miRNAs affecting numerous GO terms related to tissue repair/regeneration processes (Figure 5). Interestingly, compared to the treatment with IFN-γ, the 3D priming appears to be more effective in both cells and EXOs in inducing an up-regulation of functional miRNAs, which regulate angiogenic pathways (Figure 4a,b and Figure 5). Moreover, the EXOs derived from both types of primed hAMSCs compared with the parental cells were mainly related to GO terms linked to regulation of the immune system (Figure 4c,d and Figure 5). These data indicate that diverse priming strategies may have a dissimilar impact on the therapeutic functions of MSCs, and the effect might be, in part, mediated by miRNAs. Thus, compared to MSCs, EXOs derived from both types of priming might be more useful as a therapeutic tool in specific immune-related diseases, while 3D priming might mainly enable pro-angiogenic functions in MSCs.

Another important aspect we highlighted in this study was the different expression patterns of miRNAs for the same priming strategy between the cells and the secreted EXOs. Indeed, though we found that both 3D culture and IFN-γ treatment induce an overexpression of functional miRNAs in EXOs, we observed different miRNA expression patterns in the cells compared with their EXOs. This phenomenon might be due to the fact that the production/maturation phases of miRNAs are likely regulated through an independent mechanism [53] not affected by some priming strategies.

It has been shown that miRNA expression may serve as a signature of cell identity through the expression of distinctive miRNA profiles [54]. However, this would appear to be difficult to apply principally to exosomal miRNAs. Our study found that priming has the potential to improve the therapeutic phenotype of both MSCs and their respective EXOs in terms of functional miRNAs, and distinct priming strategies might enhance different therapeutic properties of MSCs. However, further studies on the regulation of both intracellular and exosomal miRNAs are needed.

## 5. Conclusions

The primary objective of this study was to explore the influence of cell priming on the modulation of MSC miRNA expression, leading to improvements in their therapeutic effects. We demonstrated that the priming of hAMSCs through 3D culture or IFN-γ treatment resulted in significant alterations in both the intracellular and exosomal miRNA profiles. Furthermore, our GO analysis indicated that priming might enhance the therapeutic properties of hAMSCs and their extracellular vesicles (EXOs) in a priming-dependent manner.

Though no correlation was found between intracellular and exosomal miRNA expression, we observed enhanced immunoregulatory and angiogenic effects in both cells and EXOs, indicating improved therapeutic properties. These findings hold the potential for the application of MSC-based products in the field of regenerative medicine. The insights gained from our study may contribute to advancing MSC-based therapies for future clinical treatments. Further investigations are needed to explore the effects of different priming approaches on MSCs that might also arise from alternative sources. This new information might be useful for fine-tuning MSC properties in a priming-dependent manner, offering new possibilities to enhance the therapeutic effectiveness of MSCs and guide their therapeutic effects towards targeted pathologies, particularly in terms of functional miRNA production.

## Figures and Tables

**Table 1:** Functional Enrichment Analysis of the DEMs.

3D MSCs		3D MSC EXOs		γ-MSCs		γ-MSCs EXOs	
GO Terms	miRNAs	GO Terms	miRNAs	GO Terms	miRNAs	GO Terms	miRNAs
response to hypoxia	miR-100-5p; miR-101-3p; miR-132-3p; miR-144-3p; miR-148a-3p; miR-181c-5p; miR-186-5p; miR-199a-5p; miR-212-3p; miR-23b-3p; miR-296-3p; miR-429	blood vessel development	let-7a-5p; let-7c-5p; miR-132-3p; miR-200c-3p; miR-212-3p; miR-214-3p; miR-21-5p; miR-22-3p; miR-34a-5p	immune system process	miR-492	apoptotic signaling pathway	miR-133b; miR-135a-5p; miR-141-3p; miR-486-3p; miR-139-5p; miR-182-5p; miR-133a-3p; miR-429; miR-448; miR-205-5p; miR-198; let-7b-5p; miR-125b-5p
response to growth factor	miR-101-3p; miR-124-3p; miR-132-3p; miR-1-3p; miR-144-3p; miR-148a-3p; miR-181c-5p; miR-183-5p; miR-186-5p; miR-194-5p; miR-199a-5p; miR-23b-3p; miR-429; miR-497-5p	hepatic Immune response	let-7a-5p; let-7c-5p	positive regulation of programmed cell death	miR-133b	morphogenesis of a branching epithelium	miR-33b-5p; miR-133b; miR-429; miR-448; miR-205-5p; miR-198; miR-125b-5p
cellular response to cytokine stimulus	miR-101-3p; miR-124-3p; miR-132-3p; miR-1-3p; miR-144-3p; miR-148a-3p; miR-183-5p; miR-186-5p; miR-194-5p; miR-199a-5p; miR-212-3p; miR-429; miR-892b	tube morphogenesis	let-7a-5p; miR-130b-3p; miR-132-3p; miR-200c-3p; miR-212-3p; miR-21-5p; miR-22-3p; miR-33b-5p; miR-34a-5p	intrinsic apoptotic signaling pathway	miR-188-3p	chronic inflammatory response	miR-452-5p; miR-125b-5p
sprouting angiogenesis	miR-101-3p; miR-124-3p; miR-132-3p; miR-144-3p	vasculature development	let-7a-5p; let-7c-5p; miR-200c-3p; miR-212-3p; miR-214-3p; miR-21-5p; miR-22-3p; miR-34a-5p	lymphocyte activation	miR-139-5p	positive regulation of response to oxidative stress	miR-452-5p; miR-125b-5p
apoptotic process	miR-100-5p; miR-101-3p; miR-124-3p; miR-1-3p; miR-148a-3p; miR-149-5p; miR-181c-5p; miR-183-5p; miR-193b-3p; miR-199a-5p; miR-212-3p; miR-23b-3p; miR-296-3p; miR-429; miR-497-5p; miR-518c-3p	apoptotic signaling pathway	let-7b-5p; miR-106b-5p; miR-15b-5p; miR-200c-3p; miR-212-3p; miR-214-3p; miR-21-5p; miR-22-3p; miR-34a-5p; miR-744-5p	cell differentiation	miR-1249-3p; miR-125a-5p; miR-125b-5p; miR-133a-3p; miR-133b; miR-135b-5p; miR-145-5p; miR-19a-3p; miR-204-5p; miR-20a-5p; miR-210-3p; miR-224-5p; miR-27b-3p; miR-331-3p; miR-335-5p; miR-33a-5p; miR-378a-3p; miR-495-3p; miR-518c-3p; miR-9-5p	epithelial to mesenchymal transition	miR-182-5p; miR-429; miR-205-5p; miR-452-5p
negative regulation of cell cycle	miR-101-3p; miR-124-3p; miR-144-3p; miR-148a-3p; miR-149-5p; miR-183-5p; miR-186-5p; miR-193b-3p; miR-212-3p; miR-219a-5p; miR-23b-3p; miR-429; miR-615-3p	tube development	miR-130b-3p; miR-132-3p; miR-200c-3p; miR-212-3p; miR-21-5p; miR-22-3p; miR-33b-5p; miR-34a-5p	regulation of phosphatidylinositol 3-kinase signaling	miR-125a-5p; miR-125b-5p; miR-133b; miR-145-5p	activation of MAPK activity	miR-205-5p; miR-125b-5p
activation of NF-kappaB-inducing kinase activity	miR-875-5p; miR-892b	positive regulation of immune system process	miR-132-3p; miR-200c-3p; miR-214-3p; miR-21-5p; miR-22-3p; miR-33b-5p; miR-34a-5p; miR-34b-5p	cellular response to growth factor stimulus	miR-125a-5p; miR-125b-5p; miR-133a-3p; miR-133b; miR-135b-5p; miR-145-5p; miR-15a-5p; miR-194-5p; miR-195-5p; miR-19a-3p; miR-204-5p; miR-23b-3p; miR-25-3p; miR-27b-3p; miR-378a-3p; miR-424-5p; miR-495-3p; miR-9-5p	T-helper cell differentiation	miR-33b-5p; miR-9-5p
positive regulation of T cell cytokine production	miR-875-5p; miR-892b	regulation of fibroblast proliferation	miR-130b-3p; miR-132-3p; miR-200c-3p; miR-212-3p; miR-21-5p; miR-22-3p; miR-34a-5p; miR-34b-5p	positive regulation of cell growth	miR-125a-5p; miR-125b-5p; miR-133a-3p; miR-133b; miR-145-5p; miR-224-5p	T cell differentiation involved in immune response	miR-33b-5p; miR-9-5p
tube development	miR-101-3p; miR-124-3p; miR-132-3p; miR-1-3p; miR-144-3p; miR-148a-3p; miR-199a-5p; miR-212-3p; miR-219a-5p; miR-335-5p; miR-429	activation of immune response	miR-132-3p; miR-15b-5p; miR-200c-3p; miR-21-5p	response to wounding	miR-125a-5p; miR-133a-3p; miR-133b; miR-145-5p; miR-194-5p; miR-195-5p; miR-19a-3p; miR-204-5p; miR-224-5p; miR-494-3p; miR-9-5p	coronary vasculature development	miR-9-5p; miR-503-5p; let-7c-5p
regulation of cell migration involved in sprouting angiogenesis	miR-101-3p; miR-124-3p; miR-132-3p; miR-1-3p; miR-144-3p; miR-497-5p	activation of innate immune response	miR-130b-3p; miR-132-3p; miR-200c-3p; miR-21-5p	anatomical structure morphogenesis	miR-125a-5p; miR-125b-5p; miR-133a-3p; miR-133b; miR-135b-5p; miR-145-5p; miR-195-5p; miR-19a-3p; miR-204-5p; miR-210-3p; miR-224-5p; miR-27b-3p; miR-331-3p; miR-335-5p; miR-33a-5p; miR-424-5p; miR-452-5p; miR-518c-3p; miR-9-5p	anatomical structure morphogenesis	miR-33b-5p; miR-9-5p; miR-1-3p; miR-133b; miR-135a-5p; miR-141-3p; miR-582-5p; miR-182-5p; miR-183-5p; miR-133a-3p; miR-429; miR-448; miR-205-5p; miR-452-5p; miR-125b-5p
programmed cell death	miR-100-5p; miR-101-3p; miR-124-3p; miR-1-3p; miR-148a-3p; miR-149-5p; miR-181c-5p; miR-183-5p; miR-193b-3p; miR-199a-5p; miR-212-3p; miR-296-3p; miR-429; miR-497-5p; miR-518c-3p	innate immune response-activating signal transduction	miR-130b-3p; miR-132-3p; miR-200c-3p; miR-21-5p	tube morphogenesis	miR-125a-5p; miR-125b-5p; miR-133a-3p; miR-133b; miR-135b-5p; miR-145-5p; miR-19a-3p; miR-204-5p; miR-210-3p; miR-224-5p; miR-27b-3p; miR-335-5p; miR-452-5p; miR-9-5p	cytokine-mediated signaling pathway	miR-9-5p; miR-1-3p; miR-98-5p; miR-133b; miR-135a-5p; miR-139-5p; miR-183-5p; miR-133a-3p; miR-429; miR-448; miR-205-5p; miR-452-5p; miR-125b-5p
tissue morphogenesis	miR-101-3p; miR-124-3p; miR-1-3p; miR-144-3p; miR-148a-3p; miR-186-5p; miR-199a-5p; miR-219a-5p; miR-410-3p; miR-429	regulation of innate immune response	miR-130b-3p; miR-132-3p; miR-200c-3p; miR-21-5p	cell activation involved in immune response	miR-125b-5p; miR-133a-3p; miR-133b	B cell homeostasis	miR-429; miR-125b-5p
negative regulation of cell motility	miR-101-3p; miR-124-3p; miR-1-3p; miR-144-3p; miR-148a-3p; miR-429	response to growth factor	let-7c-5p; miR-130b-3p; miR-132-3p; miR-15b-5p; miR-200c-3p; miR-214-3p; miR-21-5p; miR-22-3p; miR-33b-5p; miR-34a-5p; miR-34b-5p; miR-424-5p; miR-744-5p	cell differentiation involved in embryonic placenta development	miR-125a-5p; miR-125b-5p; miR-133b	lymphocyte homeostasis	miR-429; miR-125b-5p
negative regulation of cellular component movement	miR-101-3p; miR-124-3p; miR-1-3p; miR-144-3p; miR-148a-3p; miR-429	response to cytokine	miR-132-3p; miR-15b-5p; miR-200c-3p; miR-212-3p; miR-214-3p; miR-21-5p; miR-22-3p; miR-33b-5p; miR-34a-5p; miR-34b-5p; miR-744-5p	leukocyte activation involved in immune response	miR-125b-5p; miR-133a-3p; miR-133b	monocyte differentiation	miR-429; miR-205-5p
positive regulation of epithelial cell migration	miR-101-3p; miR-124-3p; miR-132-3p; miR-1-3p; miR-144-3p; miR-149-5p; miR-199a-5p; miR-429	insulin receptor signaling pathway	miR-15b-5p; miR-200c-3p; miR-21-5p; miR-22-3p	leukocyte mediated immunity	miR-10a-5p; miR-133a-3p; miR-133b	chemokine C-C motif ligand 2 secretion	miR-9-5p; miR-1-3p
positive regulation of cell migration involved in sprouting angiogenesis	miR-101-3p; miR-124-3p; miR-132-3p; miR-144-3p; miR-199a-5p	regulation of endothelial cell migration	miR-132-3p; miR-200c-3p; miR-21-5p; miR-22-3p	cytokine-mediated signaling pathway	miR-125a-5p; miR-125b-5p; miR-133a-3p; miR-133b; miR-135b-5p; miR-145-5p; miR-15a-5p; miR-204-5p; miR-224-5p; miR-23b-3p; miR-378a-3p; miR-452-5p; miR-9-5p	coronary vasculature morphogenesis	miR-9-5p; miR-1-3p
cellular response to fibroblast growth factor stimulus	miR-124-3p; miR-132-3p; miR-1-3p	negative regulation of apoptotic process	let-7a-5p; let-7c-5p; miR-15b-5p; miR-200c-3p; miR-212-3p; miR-214-3p; miR-21-5p; miR-22-3p; miR-30e-3p; miR-33b-5p; miR-34a-5p; miR-543; miR-744-5p	regulation of apoptotic process	miR-106b-5p; miR-125a-5p; miR-125b-5p; miR-133a-3p; miR-133b; miR-145-5p; miR-15a-5p; miR-195-5p; miR-19a-3p; miR-204-5p; miR-20a-5p; miR-210-3p; miR-27b-3p; miR-331-3p; miR-33a-5p; miR-378a-3p; miR-424-5p; miR-495-3p; miR-518c-3p; miR-9-5p	developmental cell growth	miR-9-5p; miR-1-3p
anatomical structure formation involved in morphogenesis	miR-101-3p; miR-124-3p; miR-1-3p; miR-144-3p; miR-148a-3p; miR-199a-5p; miR-29b-1-5p; miR-335-5p; miR-429	cellular response to cytokine stimulus	miR-132-3p; miR-15b-5p; miR-200c-3p; miR-212-3p; miR-214-3p; miR-21-5p; miR-22-3p; miR-33b-5p; miR-34a-5p; miR-744-5p	positive regulation of cell differentiation	miR-125a-5p; miR-125b-5p; miR-133b; miR-135b-5p; miR-145-5p; miR-19a-3p; miR-204-5p; miR-224-5p; miR-23b-3p; miR-329-3p; miR-518c-3p; miR-9-5p	fibroblast migration	miR-33b-5p; miR-1-3p
mesenchyme development	miR-101-3p; miR-124-3p; miR-144-3p; miR-148a-3p; miR-199a-5p; miR-219a-5p; miR-29b-1-5p; miR-410-3p; miR-429	cellular response to oxygen levels	miR-106b-5p; miR-132-3p; miR-200c-3p; miR-212-3p; miR-214-3p; miR-21-5p; miR-22-3p; miR-33b-5p; miR-34a-5p; miR-543	positive regulation of endothelial cell proliferation	miR-125a-5p; miR-133b; miR-145-5p	positive regulation of MAP kinase activity	miR-503-5p; miR-429; miR-205-5p; miR-125b-5p
fibroblast migration	miR-101-3p; miR-1-3p; miR-144-3p	lymphocyte activation	miR-15b-5p; miR-200c-3p; miR-214-3p; miR-21-5p; miR-22-3p; miR-34a-5p; miR-34b-5p	positive regulation of vascular endothelial cell proliferation	miR-125a-5p; miR-133b; miR-145-5p	leukocyte differentiation	miR-9-5p; miR-133b; miR-182-5p; miR-133a-3p; miR-429; miR-205-5p; miR-125b-5p

## Data Availability

The raw data supporting the conclusions of this article will be made available by the authors without undue reservation to any qualified researcher on reasonable request.
